# The impact of iodine supplementation and bread fortification on urinary iodine concentrations in a mildly iodine deficient population of pregnant women in South Australia

**DOI:** 10.1186/1475-2891-12-32

**Published:** 2013-03-15

**Authors:** Vicki L Clifton, Nicolette A Hodyl, Paul A Fogarty, David J Torpy, Rachel Roberts, Ted Nettelbeck, Gary Ma, Basil Hetzel

**Affiliations:** 1 Robinson Institute, University of Adelaide, Adelaide, SA, Australia; 2 School of Psychology, University of Adelaide, Adelaide, SA, Australia; 3 Department of Endocrinology, University of Adelaide, Adelaide, SA, Australia; 4 Department of Medicine, University of Western Sydney, Campbelltown, NSW, Australia; 5 International Council for Control of Iodine Deficiency Disorders Global Network (ICCIDD), Westmead Hospital, Westmead, NSW, Australia; 6 Robinson Institute, Lyell McEwin Hospital, Haydown Rd, Elizabeth Vale, SA, 5112, Australia

**Keywords:** Iodine, Pregnancy, Urine, Supplements

## Abstract

Mild iodine deficiency during pregnancy can have significant effects on fetal development and future cognitive function. The purpose of this study was to characterise the iodine status of South Australian women during pregnancy and relate it to the use of iodine-containing multivitamins. The impact of fortification of bread with iodized salt was also assessed. Women (n = 196) were recruited prospectively at the beginning of pregnancy and urine collected at 12, 18, 30, 36 weeks gestation and 6 months postpartum. The use of a multivitamin supplement was recorded at each visit. Spot urinary iodine concentrations (UIC) were assessed. Median UICs were within the mildly deficient range in women not taking supplements (<90 μg/L). Among the women taking iodine-containing multivitamins UICs were within WHO recommendations (150–249 μg/L) for sufficiency and showed an increasing trend through gestation. The fortification of bread with iodized salt increased the median UIC from 68 μg/L to 84 μg/L (p = .011) which was still in the deficient range. Pregnant women in this region of Australia were unlikely to reach recommended iodine levels without an iodine supplement, even after the mandatory iodine supplementation of bread was instituted in October 2009.

## Introduction

Iodine deficiency affects nearly 2 billion people globally and is easily preventable with the use of iodized salt in the diet [1]. Iodine deficiency during pregnancy is associated with increased rates of stillbirths, spontaneous abortions, and congenital anomalies [2] and is the leading worldwide preventable cause of intellectual impairment in children [3]. A global survey of iodine sufficiency in 2011 identified that although there was some improvement worldwide in iodine sufficiency, 29.8% of school aged children still had insufficient iodine intake [4]. The iodine intake of pregnant women in Australia has been found to be inadequate in several studies over the past decade [5-11] but has not been examined in the South Australian population where this current study has been conducted. As a result of the studies that have been conducted in Australia, the National Health and Medical Research Council (NHMRC) has recommended that all women who are pregnant, breastfeeding or considering pregnancy, with the exception of women with pre-existing thyroid conditions who should seek advice from their medical practitioner, are to take a daily iodine supplement of 150 micrograms per day [12].

To increase the iodine intake of the Tasmanian population, which has a well-documented history of endemic iodine deficiency [13], a trial of voluntary iodine fortification of bread was commenced in 2001. There has been a significant improvement in the iodine status of Tasmanian schoolchildren from mildly iodine-deficient to borderline iodine-sufficient [14]. However, the iodine status of pregnant Tasmanian women showed a non-significant improvement rising from 76 to 81–86 μg/L, still in the mildly deficient range [15].

The National Iodine Nutrition Study [16] confirmed the existence of an inadequate iodine intake in the mainland Australian population and mandatory iodine fortification of bread was introduced in October 2009 in all Australian States and Territories. This study assessed the iodine status of pregnant South Australian women throughout gestation in relation to their reported intake of dietary iodine-containing multivitamins and the introduction of mandatory iodine fortification of bread in Australia.

Our aim was to examine iodine status in pregnancy in a new region of Australia examining the effect of the recommendation for routine iodine supplementation.

## Methods

### Study participants

The study was approved by the Queen Elizabeth and Lyell McEwin Hospitals Human Research Ethics Committee as well as the University of Adelaide Human Research Ethics Committees. Pregnant women (n = 196), aged 17–38 years were recruited at the Lyell McEwin Hospital antenatal clinic between January 2009 and July 2010, during the first trimester of pregnancy and provided written informed consent for participation. This hospital is located in a region of socio-economic disadvantage, as characterised by a relative social economic index for areas (SEIFA) score in the lowest decile for Australia [17]. A urine sample was collected at the clinic visit at 12, 18, 30 and 36 weeks gestation. 126 women (64%) provided at least 3 samples, while 24 women (12%) provided only 1 sample, due to non-attendance at their scheduled antenatal appointments. Removal of these data points does not alter the findings of this study and so the samples were included. At each visit women were asked if they were taking a daily dietary multivitamin during pregnancy and the brand was noted. Dietary iodine-containing multivitamins were used by 47% (n = 92) of women during pregnancy, while 23% (n = 45) of women used no multivitamins and 30% (n = 59) used dietary multivitamins that did not contain iodine. Those women who used no multivitamins or a multivitamin that did not contain iodine during pregnancy were grouped because their UIC were not significantly different. It was predicted that urinary iodine concentration (UIC) would increase by 54 μg/ml with bread consumption and an iodine containing multivitamin contains 75 μg with a recommended dose of 2 tablets per day. Those women with complications such as thyroid disease, pre-eclampsia, gestational diabetes, infection or preterm delivery were excluded from the analysis.

### Urinary iodine concentration

Urinary iodine was assayed in duplicate using a previously published method [18]. In brief, spot urinary iodine concentrations were determined by a Sandell-Kolthoff reaction. Substances known to interfere with the sensitive colorimetry of the Sandell-Kolthoff reaction, such as thiocyanate, were removed by an ammonium persulphate digestion. Sensitivity of the assay is 5 μg/L. The inter-assay co-efficient of variations (%CV) at 85 μg/L; 185 μg/L and 320 μg/L were 6%; 5% and 8% respectively.

### Statistical analyses

Data were analysed using the Statistical Package for the Social Sciences (SPSS v 17). Clinical characteristics were compared according to the use of iodine-containing multivitamins using t-tests (normally distributed data) or Mann Whitney U-tests (non-normally distributed data). The non-parametric Friedman’s χ^2^ test was used to assess the UIC data over gestation, while Mann Whitney U tests were used to compare UIC data according to the use of iodine-containing multivitamins. Chi-square analysis was used to assess the frequency of UIC over 150 μg/L with the use of iodine-containing multivitamins.

## Results

Maternal age, weight (at 12 weeks gestation), body mass index and parity were not significantly different between those women who used iodine-containing multivitamins during pregnancy and those who did not (Table 1). Women who used iodine-containing multivitamins were significantly taller compared to the women who did not used iodine-containing multivitamins, or who used no supplements during pregnancy (p = 0.04). Smoking rates were not different between the supplement and no supplement groups (Table 1), and smoking was not associated with changes in urinary iodine concentrations (data not shown).

**Table 1:** Clinical characteristics of the 196 pregnant women included in the study according to their use of iodine-containing multivitamins during pregnancy

	**No supplements n = 45 (23%)**	**Non-iodine-containing multivitamins n = 58 (30%)**	**Iodine-containing multivitamins n = 93 (47%)**	**p value**
Age (years)	24 (22–30)	25 (23–29)	26 (22–30)	0.39
Height (cm)	162 (157–167)	162 (159–166)	165 (161–168)	0.04
Weight (12 weeks) (kg)	77 (64–92)	67 (56–94)	71 (62–81)	0.28
BMI (12 weeks)	29 (24–32)	23 (21–31)	26 (23–30)	0.11
Parity, med (min-max)	1 (0–5)	1 (0–4)	1 (0–3)	0.41
Cigarettes use, n (%)	14 (31)	7 (12)	11 (12)	0.12

Data are presented as median (25^th^-75^th^ centiles) unless otherwise indicated.

**Figure 1:**
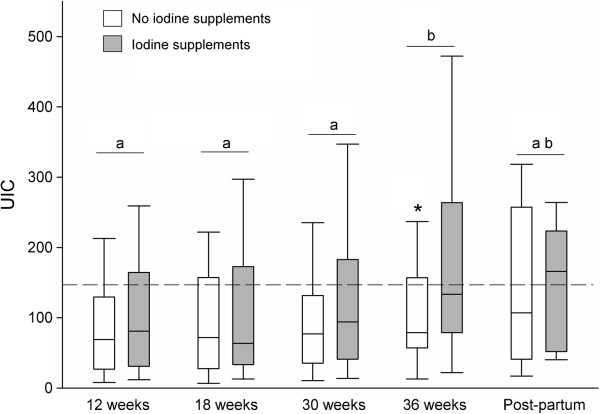
**Urinary iodine concentrations during pregnancy and 6 months postpartum according to the use of iodine-containing multivitamins, measured at 12 weeks (no iodine-containing multivitamins n = 89, iodine-containing multivitamins n = 76), 18 weeks (no iodine-containing multivitamins n = 81, iodine-containing multivitamins n = 79), 30 weeks (no iodine-containing multivitamins n = 58, iodine-containing multivitamins n = 62) 36 weeks (no iodine-containing multivitamins n = 51, iodine-containing multivitamins n = 60) and at 6 months post-partum (no iodine-containing multivitamins n = 21, iodine-containing multivitamins n = 17).** Line and boxes represent the median and the inter-quartile range and whiskers represent the 95% confidence interval. *p < 0.05 between iodine supplement and no supplement group at 36 weeks (Mann Whitney U test); significant differences across gestation are represented by different alpha symbols (p < 0.01).

Urinary iodine concentrations increased significantly as pregnancy progressed when all groups were combined (Friedman’s χ2 = 12.974, p = 0.005). Post hoc comparisons indicated that UIC at 36 weeks (median = 118) was significantly higher than UIC at 12 (median = 73), 18 (median = 68) and 30 weeks gestation (median = 84; p < 0.025 in all instances, Figure 1).

The effect of dietary iodine-containing multivitamins was assessed at each gestational time-point and 6 months post-partum. As UIC did not differ between women consuming dietary supplements with no iodine and women who consumed no supplements during pregnancy, these women were grouped together for the purpose of analysis. The consumption of dietary supplements containing iodine significantly increased UIC at 36 weeks gestation and appeared to be sustained in the postpartum period (Mann Whitney U = 1085, p = 0.008; Figure 1).

The proportion of women with UIC above the WHO recommendations of 150 μg/L in pregnancy was assessed according to the use of supplementation at each time point. At 30 and 36 weeks gestation, significantly more women who used iodine containing dietary supplements were above the WHO recommended guidelines for pregnant women of 150 μg/L compared to women who did not use iodine containing dietary supplements (Table 2).

**Table 2:** Number of women with UIC ≥ 150 μg/L at each gestational time point according to the consumption of iodine-containing dietary supplements

	**No multivitamin**	**Iodine containing multivitamin**	**p**
12 weeks	16 (18%)	21 (28%)	0.09
	Total n = 89	Total n = 76	
18 weeks	23 (28%)	21 (27%)	0.47
	Total n = 81	Total n = 79	
30 weeks	**11 (19%)**	**23 (37%)**	**0.022**
	Total n = 58	Total n = 62	
36 weeks	**13 (25%)**	**26 (43%)**	**0.038**
	Total n = 51	Total n = 60	
Post-partum	9 (45%)	9 (53%)	0.39
	Total n = 21	Total n = 17	

Data are presented at each time point as n (%) of women who provided a spot urine sample at each appointment. Significance level represents result of Chi squared analysis at each gestational time point.

The median UIC for pregnant women was 82 μg/L (with minimum-maximum 1–754 μg/L) indicating mild iodine deficiency. The consumption of iodine supplements was associated with a significantly higher UIC over pregnancy (median = 89ug/L, minimum-maximum 1–754 μg/L) than women who did not consume iodine-containing multivitamins (median = 75ug/L, minimum-maximum 1–476 ug/L; p = 0.003 Mann Whitney U test). Overall, there was an improvement in iodine status associated with the mandatory iodine fortification of bread. The median UIC of pregnant women not using an iodine-containing multivitamin during pregnancy was 68 μg/L (range 10–327 μg/L, n = 84 samples) prior to bread fortification, and significantly increased to 84 μg/L (range 83.4–393 μg/L, n = 94 samples) post-intervention (*p* = 0.01).

## Discussion

This is the first South Australian study to examine iodine sufficiency during pregnancy, encompassing the period where mandatory fortification of bread and cereal with iodized salt was implemented, to simultaneously examine the effect of the recently recommended iodine-containing multivitamins during pregnancy. The data indicates that overall women in the Elizabeth-Playford Region of Adelaide are mildly iodine deficient in the absence of supplementation but iodine-containing multivitamins designed for pregnancy lead to iodine sufficiency as measured by UIC (>150 ug/L). Furthermore the fortification of bread significantly increased UIC but not to a level compatible with iodine sufficiency, suggesting that supplements are still required despite the iodized salt initiative in bread and cereal production. Overall these data suggest that women should consider using an iodine-containing multivitamin during pregnancy to improve their iodine concentrations.

Previous studies conducted in other states of Australia indicate that the national population of pregnant women are mildly iodine deficient. Hamrosi et al [8] examined median UIC in Victorian women of differing ethnicities and reported that all groups were mildly iodine deficient with only slight variations in median concentrations which may be accounted for by dietary differences. More recently it was reported in the Gippsland region of Victoria that bread fortification with iodised salt failed to improve UIC of pregnant women [19]. Mackerras et al. [20] reported mild iodine deficiency in Aboriginal populations of the Northern Territory and determined bread fortification was not sufficient for pregnant and non-pregnant individuals of this population. In NSW the median UIC was 85 ug/L in pregnant women [6,7] and this is comparable to the South Australian data reported in the current study. Charlton et al [5,6] reported that 35% of pregnant women used iodine-containing multivitamins during pregnancy which significantly increased their UIC and placed them in the sufficient iodine range. In Tasmania the mandatory fortification of bread has improved the UIC but levels remain in the mildly deficient range for pregnant women [14]. These data indicate Australian women are iodine deficient and this deficiency is not resolved in pregnancy by mandatory fortification of bread with iodised salt. A multivitamin containing iodine for pregnancy and lactation appears to be the only way to resolve deficiency at this crucial time in fetal and child development.

Poor maternal iodine status during pregnancy has been shown to be associated with alterations in the neonatal thyroid function. McElduff et al. [9] identified that in two populations of neonates 5–8% of the neonates had mild thyroid deficiency as indicated by a thyroid stimulating hormone (TSH) concentrations of greater than 5 mlU/L. More recently Rahman et al [21] reports that neonatal TSH levels of greater than 5 mlU/L have increased by 5% in the last 5 years. Travers et al [7] reported that mothers with a UIC of less than 50 ug/L were 2.6 times more likely to have a neonate with mild thyroid deficiency. A Swiss study has shown that neonates and infants are especially susceptible to iodine deficiency if breast fed and not receiving food or formula fortified with iodine [22]. Hence, mild iodine deficiency during pregnancy and in the neonatal period can have significant effects on childhood development and cognitive function.

Previous studies have reported in iodine sufficient populations of Japan and Mexico, that UIC decreased with pregnancy [23,24] and was further decreased postpartum [24]. Similar gestational decreases of UIC were reported in iodine deficient pregnant population of India [25]. We observed a slight rise in iodine in the group of women taking an iodine-containing multivitamin as gestation progressed and a postpartum decrease in UIC. The range of UIC in the population was highly variable. This may be due to variations in dietary intakes within this population despite similarities in age, weight and location of residence. However, high variability in UIC is seen in all studies making this measure only useful in populations rather than in individual assessment of iodine sufficiency. The discrepancy in these findings does not detract from the evidence that pregnant South Australian women are mildly iodine deficient.

The benefits of iodine sufficiency are well known but poor knowledge of the impact of iodine deficiency by the Australian community, along with a lack of nutritional education and supplementation means that pregnant women and their children remain at risk of iodine deficiency. Our current study identifies that South Australian women from a socially disadvantaged background are mildly deficient and could benefit from supplementation during pregnancy and while breast feeding.

## Competing interests

The authors declare that they have no competing interests.

## Authors’ contributions

VC designed the study, supervised the work and wrote the paper; NA analysed the data and edited the paper; PF; collected the samples; DT, RR, TN, BH had intellectual input into data analysis and the writing of the paper; GM measured the urinary iodines. All authors read and approved the final manuscript.
